# Short-term outcomes of ovarian vein embolization with adjunctive local sclerotherapy in patients with pelvic venous disorder and vulvar varicosities: a single-center retrospective experience

**DOI:** 10.3389/fsurg.2026.1787709

**Published:** 2026-06-24

**Authors:** Kai Zheng, Qiangqiang Nie, Bin Ni, Xixi Guo, Yuguang Yang, Peng Liu, Zhidong Ye, Bo Ma, Xueqiang Fan

**Affiliations:** 1 China-Japan Friendship Hospital (Institute of Clinical Medical Sciences), Chinese Academy of Medical Sciences & Peking Union Medical College, Beijing, China; 2 Department of Cardiovascular Surgery, China-Japan Friendship Hospital, Beijing, China; 3 China-Japan Friendship Hospital (Institute of Clinical Medical Sciences), Beijing, China

**Keywords:** endovenous treatment, external genital varicosities, ovarian vein embolization, pelvic venous disorder, sclerotherapy, vulvar varicosities

## Abstract

**Objective:**

To describe the short-term safety, procedural characteristics, and clinical outcomes of ovarian vein embolization with adjunctive local sclerotherapy in a selected subgroup of women with pelvic venous disorder presenting with vulvar/external genital varicosities.

**Methods:**

This retrospective single-center experience included 16 women with pelvic venous disorder presenting with concomitant vulvar/external genital varicosities who were treated between 2019 and 2023. The treatment strategy was primarily directed at pelvic venous reflux with ovarian vein embolization, followed by adjunctive local sclerotherapy for symptomatic vulvar/external genital varicosities during the same interventional session. Clinical assessment included symptom evaluation, visual analog scale (VAS), pelvic venous clinical severity score (PVCSS), and follow-up imaging.

**Results:**

The median follow-up duration was 7.0 months (IQR, 6.0–8.5 months). Technical success was achieved in all patients. Mean VAS decreased from 5.8 ± 1.8 before treatment to 0.9 ± 0.9 at follow-up, and mean PVCSS decreased from 10.7 ± 2.3 to 4.0 ± 1.6. Complete symptom relief was observed in 9 patients (56%) and partial relief in 7 patients (44%). No patient required reintervention during follow-up, and no major procedure-related adverse events were observed.

**Conclusions:**

Ovarian vein embolization with adjunctive local sclerotherapy was technically feasible and was associated with short-term symptom improvement in women with pelvic venous disorder presenting with vulvar/external genital varicosities.

## 1 Introduction

Pelvic venous disorder (PeVD) is a heterogeneous venous condition in women characterized by pelvic venous reflux, venous obstruction, or both. Its clinical presentation is variable and may include chronic pelvic pain, dyspareunia, urinary symptoms, and vulvar or lower-extremity varicosities (1, 2). Vulvar varicosities (VV) represent an important manifestation of pelvic venous disease and may be associated with underlying pelvic venous reflux (3).

Endovascular embolization is an established treatment option for selected patients with symptomatic PeVD, and recent systematic reviews and larger retrospective studies have reported favorable technical and clinical outcomes in the overall PeVD or pelvic congestion syndrome population (4–6). However, less attention has been paid to women presenting with concomitant vulvar or external genital varicosities as a more specific clinical subgroup (3). In these patients, both treatment of pelvic venous reflux and local management of symptomatic varicosities may be clinically relevant, but the practical integration of ovarian vein embolization and adjunctive local sclerotherapy, as well as the short-term outcomes of this embolization-based strategy in this specific subgroup, remain insufficiently described in focused real-world series.

Therefore, this retrospective single-center case series aimed to describe the short-term safety, procedural characteristics, and clinical outcomes of ovarian vein embolization with adjunctive local sclerotherapy in women with PeVD and concomitant vulvar/external genital varicosities. We hypothesized that this treatment strategy would be technically feasible and associated with short-term symptom improvement in this specific clinical subgroup.

## 2 Materials and methods

### 2.1 Study design and population

This retrospective single-center case series was approved by the Clinical Research Ethics Committee of China-Japan Friendship Hospital (2024-KY-246-1). Given the retrospective design and use of de-identified clinical data, the requirement for individual informed consent for study participation was waived by the ethics committee. Written informed consent for the interventional procedures had been obtained as part of routine clinical care.

We screened female patients treated at our institution between January 2019 and December 2023 for pelvic venous disorder (PeVD) presenting with concomitant vulvar/external genital varicosities. Eligible patients were those who underwent ovarian vein embolization for pelvic venous reflux, followed by adjunctive local sclerotherapy for symptomatic vulvar/external genital varicosities during the same interventional session.

**Figure 1:**
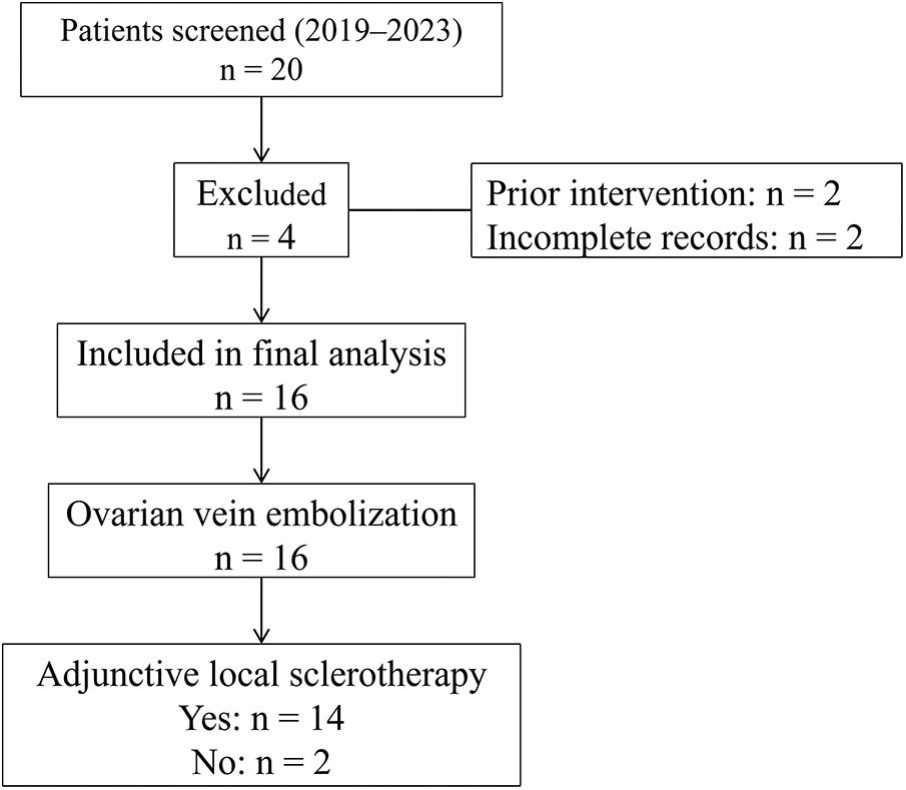
STROBE flow diagram of patient screening, exclusion, and final study cohort.

Patients were excluded if they had undergone prior pelvic or vulvar interventional treatment or surgery within 1 year before the index procedure, had received recent laser or pharmacologic treatment specifically targeting vulvar varicosities, were pregnant or planning pregnancy within the following year, or had incomplete clinical or imaging records precluding outcome assessment. Two patients were excluded because of prior intervention, and two were excluded because of incomplete records. The final analysis included 16 patients. A STROBE flow diagram was added to illustrate the number of patients screened, the reasons for exclusion, and the final analyzed cohort (Figure 1).

Clinical data were extracted from electronic medical records, procedural reports, and the picture archiving and communication system (PACS) by two experienced interventional radiologists. Recorded baseline variables included age, body mass index, major presenting complaints, associated symptoms, anatomical venous findings on imaging and venography, and procedural details. Clinical presentation and venous findings were categorized according to the Symptoms-Varices-Pathophysiology (SVP) classification for pelvic venous disorders when applicable (7). In the present study, vulvar/external genital varicosities were considered a clinical manifestation associated with PeVD rather than a primary vulvar vascular malformation. Because of the retrospective design, exclusion of alternative diagnoses such as endometriosis was based on routine clinical evaluation, gynecologic assessment, and available imaging findings documented in the medical records rather than on a prespecified study-specific diagnostic protocol.

### 2.2 Interventional procedure

All procedures were performed using a transfemoral venous approach under fluoroscopic guidance. After venous access was obtained using the Seldinger technique, diagnostic venography was performed to assess the inferior vena cava, renal veins, ovarian veins, internal iliac venous drainage, and pelvic venous reflux patterns. When identifiable on venography, reflux pathways and clinically relevant pelvic escape pathways were recorded descriptively; however, detailed mapping of specific perineal, inguinal, or clitoral reflux points was not systematically documented using a dedicated standardized mapping protocol in all patients. The presence and laterality of ovarian venous reflux were recorded. Evaluation of iliac or renal vein compression/obstruction, including possible left renal vein or common iliac vein stenosis, was based on preprocedural imaging and intraprocedural venographic findings when clinically suspected, but was not systematically assessed using a dedicated obstruction protocol in all patients.

After selective catheterization of the refluxing ovarian vein, embolization was performed using detachable or pushable coils, including Interlock detachable coils and Cook coils. Adjunctive 3% polidocanol foam was used at the operator's discretion according to venographic findings. Completion venography was performed to confirm technical occlusion of the target refluxing vein and reduction or elimination of reflux.

For patients with symptomatic vulvar/external genital varicosities, adjunctive local foam sclerotherapy with 3% polidocanol was performed during the same interventional session as ovarian vein embolization. The treatment sequence consisted of ovarian vein embolization followed by direct local treatment of the vulvar/external genital variceal cluster. Intraluminal 3% polidocanol foam during ovarian vein embolization was used selectively according to intraprocedural venographic findings. Under direct puncture of the symptomatic varicose cluster, foam sclerosant was injected slowly until adequate filling of the target varicosities was achieved. The sclerosant volume was determined according to the extent and distribution of the varicosities and operator judgment. Repeat local sclerotherapy, when performed, was based on residual symptoms and follow-up clinical and imaging findings.

### 2.3 Outcome measures and follow-up

The primary outcome of the study was short-term clinical improvement at follow-up, assessed by change in the visual analog scale (VAS) score and by overall symptom relief. Secondary outcomes included technical success, change in pelvic venous clinical severity score (PVCSS) (8), procedure-related adverse events, recurrence, need for reintervention, and interval imaging improvement when follow-up imaging was available.

Technical success was defined as successful catheterization and embolization of the intended refluxing ovarian vein(s), with satisfactory reduction or elimination of reflux on completion venography. Clinical follow-up was performed using outpatient visits and/or telephone interviews, with the last available follow-up within 6–12 months after the index procedure used for outcome assessment. This follow-up window reflected the retrospective design and the availability of routine clinical follow-up data rather than a prespecified long-term follow-up protocol. Recorded follow-up variables included VAS, PVCSS, patient-reported symptom improvement, adverse events, and the need for additional treatment.

Overall symptom relief was categorized as complete relief, partial relief, or no improvement using predefined clinical categories adapted from prior interventional literature (9). Complete relief was defined as resolution of the main presenting symptoms without the need for additional treatment during follow-up. Partial relief was defined as clinically meaningful improvement in symptoms compared with baseline without complete resolution. No improvement was defined as unchanged or worsened symptoms after treatment.

Recurrence was defined as reappearance of the patient's preprocedural symptoms after initial improvement and/or recurrent clinically evident vulvar/external genital varicosities requiring repeat treatment during follow-up.

Procedure-related adverse events included access-site complications, allergic reactions, non-target embolization, coil migration, thrombosis, post-procedural pain requiring treatment, and any unplanned reintervention. Follow-up imaging was not fully standardized because of the retrospective design. When available, ultrasound, magnetic resonance imaging, computed tomography, or repeat venography was reviewed to assess residual or recurrent pelvic reflux and interval improvement in vulvar/external genital varicosities. When magnetic resonance imaging of sufficient quality was available both before and after treatment, interval change in lesion extent could be estimated descriptively using three-dimensional measurements and an ellipsoid approximation, as previously described in the imaging literature (10). These estimates were not available in a standardized or consistently documented manner across the cohort and were therefore not reported as formal quantitative outcome data. However, because imaging modalities and follow-up protocols were not uniform across patients, and no standardized imaging measurement protocol was applied across the entire cohort, imaging findings were analyzed descriptively and were considered supportive rather than primary outcome measures.

### 2.4 Statistical analysis

Statistical analyses were performed using SPSS version 22.0 (IBM Corp., Armonk, NY, USA). Continuous variables were expressed as mean ± standard deviation or median (interquartile range), as appropriate after assessment of distribution normality. Selected skewed variables, including follow-up duration, coil counts, and polidocanol volumes, were summarized as median (interquartile range). Categorical variables were summarized as counts and percentages.

Distribution normality was assessed before selection of the statistical test. For paired comparisons between baseline and follow-up clinical scores, paired t-tests were used for normally distributed variables and Wilcoxon signed-rank tests were used when normality assumptions were not met. Two-sided *p* values < 0.05 were considered statistically significant. Given the small sample size and exploratory nature of the study, formal adjustment for multiple comparisons was not performed, and the statistical analysis was intended to support descriptive hypothesis-generating interpretation.

## 3 Results

### 3.1 Baseline clinical characteristics

The final study population included 16 women. The mean age at treatment was 42.8 ± 9.7 years (range, 26–62 years), and the mean body mass index was 23.7 ± 3.2 kg/m^2^ (range, 18.6–32.1 kg/m^2^). The cohort showed a heterogeneous symptom profile. The primary presenting complaints were abdominal or pelvic symptoms in 10 patients, including abdominal pain (*n* = 5) and abdominal heaviness (*n* = 5). Other presenting manifestations included urinary urgency (*n* = 2), perineal swelling (*n* = 2), lower-extremity varicosities (*n* = 2), vaginal mass (*n* = 1), and vaginal bleeding (*n* = 1).

Associated symptoms identified after detailed baseline history taking included dysmenorrhea in 10 patients, dyspareunia in 13, and urinary urgency in 15. In contrast to Table 1, which summarizes the primary presenting complaint at the initial visit, Table 2 summarizes associated symptoms and the distribution of varicosities recorded during the baseline clinical assessment. With respect to the anatomical distribution of varicosities, all 16 patients had vulvar varicosities, 5 had vaginal varicosities, and 6 had lower-extremity varicosities. Baseline clinical and anatomical patterns, including SVP classification, are summarized in Tables 1, 2.

**Table 1:** Baseline characteristics and SVP classification of 16 women with pelvic venous disorder and concomitant vulvar/external genital varicosities.

Patient No.	Age, years	BMI, kg/m^2^	Main presenting complaint	SVP Classification		
				S	V	P
1	41	24.1	Abdominal pain	S_2,3a_	V_2,3a_	P_LGV,R,NT_
2	35	20.8	Abdominal pain, urinary urgency	S_1,3a_	V_2,3a_	P_LGV,R,NT_
3	44	20.7	Abdominal pain	S_2,3a_	V_2,3a_	P_BGV,R,NT_
4	36	26.2	Abdominal pain, abdominal distension	S_2,3a_	V_2,3a,3b_	P_BGV,R,NT_
5	41	18.6	Urinary urgency	S_1,3a_	V_2,3a_	P_LGV,R,NT_
6	62	23.5	Abdominal distension	S_2,3a_	V_2,3a,3b_	P_LGV,R,NT_
7	38	20.6	Varicose veins of the lower extremities	S_0,3b_	V_0,3a,3b_	P_LGV,R,NT_
8	39	24.1	Abdominal distension	S_2,3a_	V_2,3a_	P_LGV,R,NT_
9	48	25.4	Abdominal distension	S_2,3a_	V_2,3a_	P_LGV,R,NT_
10	32	22.3	Abdominal pain	S_2,3a_	V_2,3a_	P_LGV,R,NT_
11	56	24.3	Abdominal distension	S_2,3a_	V_2,3a_	P_LGV,R,NT_
12	42	24.8	Vaginal bleeding	S_0,3a_	V_2,3a_	P_LGV,R,NT_
13	36	32.1	Perineal swelling	S_3a_	V_2,3a_	P_LGV,R,NT_
14	53	20.9	Varicose veins in the vagina	S_2,3a_	V_2,3a,3b_	P_LGV,R,NT_
15	55	26.1	Perineal swelling	S_2,3a_	V_2,3a_	P_LGV,R,NT_
16	26	24.5	Varicose veins of the lower extremities	S_2,3b_	V_2,3a,3b_	P_LGV,R,NT_

BMI, body mass index; SVP, Symptoms-Varices-Pathophysiology classification.

S indicates presenting symptoms; V indicates involved variceal reservoirs; *P* indicates pathophysiology, including anatomic, hemodynamic, and etiologic features.

S0, no pelvic symptoms; S1, urinary symptoms; S2, pelvic pain symptoms; S3a, vulvar symptoms/varicosities; S3b, lower-extremity varicosities.

V0, no identified variceal reservoir; V2, gonadal vein-related reservoir; V3a, vulvar/external genital varicosities; V3b, lower-extremity varicosities.

PLGV, pelvic leak point involving the left gonadal vein; PBGV, pelvic leak point involving bilateral gonadal veins; R, reflux; NT, non-thrombotic etiology.

**Table 2:** Baseline symptom profile and distribution of varicosities.

Variable	No. (%)
Dysmenorrhea	10 (62.5)
Dyspareunia	13 (81.3)
Urinary urgency	15 (93.8)
Vaginal varicosities	5 (31.3)
Vulvar varicosities	16 (100.0)
Lower-extremity varicosities	6 (37.5)

Values are presented as *n* (%). Table 2 summarizes associated symptoms and the distribution of varicosities identified after detailed baseline history taking and does not represent the primary presenting complaint at the initial visit.

### 3.2 Venographic findings and procedures

All 16 patients underwent ovarian vein embolization for pelvic venous reflux and adjunctive local sclerotherapy for symptomatic vulvar/external genital varicosities during the same interventional session. Left ovarian vein reflux was identified in all 16 patients, and 2 patients also had right ovarian vein reflux. Intraluminal 3% polidocanol foam during ovarian vein embolization was used in 14 patients, whereas 2 patients underwent ovarian vein embolization without intraluminal foam. Technical success was achieved in all patients. For ovarian vein embolization, the median number of Interlock coils used was 3 (IQR, 2–4), the median number of Cook coils used was 2 (IQR, 1–3), and the administered volume of 3% polidocanol foam was 10 mL per patient (IQR, 5–15 mL). Procedural details are summarized in Table 3.

**Table 3:** Venographic findings and procedural details.

Patient No.	Ovarian vein embolization	Ovarian vein reflux, grade (I→IV)	Interlock coils, *n*	Cook coils, *n*	Polidocanol foam for OVE, mL	Polidocanol foam for adjunctive local sclerotherapy, mL
1	Yes	Left (III)	3	1	0	1
2	Yes	Left (IV)	3	1	20	4
3*	Yes	Left (IV), Right (II)	Left (4), Right (0)	Left (1), Right (0)	Left (10), Right (5)	3
4*	Yes	Left (IV), Right (III)	Left (2), Right (4)	Left (2), Right (2)	Left (10), Right (15)	3
5	Yes	Left (II)	4	2	0	4
6	Yes	Left (III)	2	1	10	2
7	Yes	Left (III)	3	0	5	3
8	Yes	Left (II)	2	2	10	5
9	Yes	Left (IV)	3	16	5	2
10	Yes	Left (II)	3	1	5	3
11	Yes	Left (III)	2	2	10	3
12	Yes	Left (III)	2	3	15	4
13	Yes	Left (III)	3	3	10	3
14	Yes	Left (III)	2	2	5	1
15	Yes	Left (IV)	4	4	20	4
16	Yes	Left (III)	2	2	10	3

All patients underwent ovarian vein embolization and adjunctive local sclerotherapy for symptomatic vulvar/external genital varicosities. Polidocanol foam volumes for ovarian vein embolization and adjunctive local sclerotherapy are reported separately.

* Only patients 3 and 4 underwent bilateral ovarian vein treatment; all others underwent unilateral ovarian vein treatment.

For adjunctive local treatment of vulvar/external genital varicosities, the administered volume of 3% polidocanol foam was 3 mL per patient (IQR, 2.5–4 mL).

### 3.3 Clinical outcomes and follow-up

The median follow-up duration was 7.0 months (IQR, 6.0–8.5 months). Mean VAS decreased from 5.8 ± 1.8 before treatment to 0.9 ± 0.9 at follow-up. Mean PVCSS decreased from 10.7 ± 2.3 to 4.0 ± 1.6.

Complete symptom relief was observed in 9 patients (56%), and partial symptom relief in 7 patients (44%). No patient was classified as having no improvement. No patient met the study definition of recurrence during follow-up, and no patient required reintervention.

**Figure 2:**
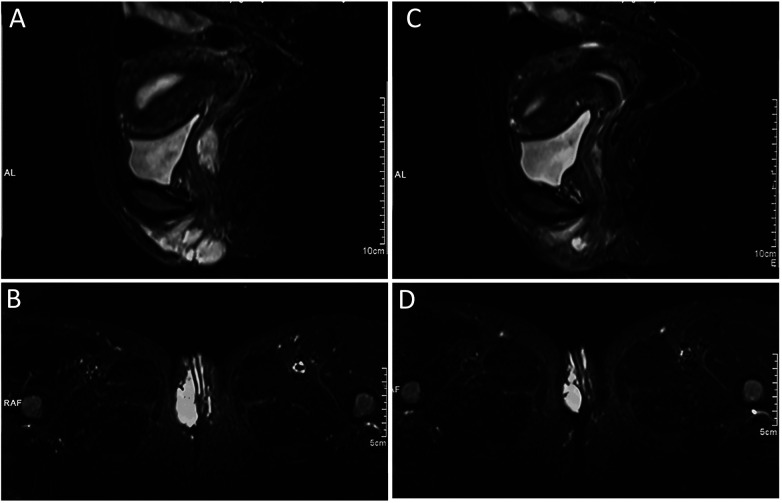
Representative preprocedural and postprocedural imaging findings in a patient with pelvic venous disorder and concomitant vulvar/external genital varicosities. **(A,B)** Preprocedural magnetic resonance imaging. **(C,D)** Postprocedural magnetic resonance imaging demonstrating interval improvement after treatment.

Follow-up imaging was available in a subset of patients and showed interval improvement in pelvic venous findings and/or vulvar/external genital varicosities; representative imaging is shown in Figure 2. Because imaging modalities and follow-up protocols were not standardized, imaging findings were interpreted descriptively.

No major procedure-related adverse events were observed during follow-up. Specifically, no access-site complications, coil migration, allergic reactions, symptomatic thrombosis, non-target embolization, post-procedural pain requiring treatment, or unplanned reintervention were recorded. Detailed follow-up data are summarized in Table 4.

**Table 4:** Clinical outcomes and follow-up findings.

Patient No.	Follow-up time, months	VAS Before (after)	PVCSS Before (after)	Symptom relief	Recurrence	Follow-up imaging modality	Descriptive imaging response
1	6	7 (1)	8 (2)	Complete	No	US, MRI	Marked
2	8	8 (2)	10 (5)	Partial	No	US, MRI	Moderate
3	12	6 (0)	11 (7)	Partial	No	US, CT	Mild
4	7	8 (3)	9 (3)	Partial	No	US, CT	Moderate
5	7	4 (0)	9 (2)	Complete	No	US	Marked
6	7	8 (1)	12 (5)	Complete	No	US	Marked
7	11	3 (0)	13 (4)	Complete	No	US	Marked
8	10	6 (1)	15 (6)	Complete	No	US, MRI	Marked
9	8	8 (2)	9 (4)	Complete	No	US	Marked
10	6	7 (2)	7 (2)	Complete	No	US	Marked
11	6	7 (1)	12 (2)	Complete	No	US, MRI	Marked
12	6	4 (0)	9 (4)	Complete	No	US	Marked
13	7	5 (1)	13 (6)	Partial	No	US	Moderate
14	9	3 (0)	11 (4)	Partial	No	US, CT	Moderate
15	6	5 (1)	14 (5)	Partial	No	US	Moderate
16	6	4 (0)	9 (3)	Partial	No	US, MRI	Moderate

VAS, visual analog scale; PVCSS, pelvic venous clinical severity score. Values are presented as baseline (follow-up).

Symptom relief was categorized as complete, partial, or no improvement.

Recurrence was defined as reappearance of preprocedural symptoms after initial improvement and/or recurrent clinically evident vulvar/external genital varicosities requiring repeat treatment during follow-up.

Because follow-up imaging modalities and protocols were not standardized, imaging findings were interpreted descriptively as mild, moderate, or marked improvement.

## 4 Discussion

In this retrospective single-center case series, ovarian vein embolization with adjunctive local sclerotherapy was technically feasible and was associated with short-term symptom improvement in women with pelvic venous disorder (PeVD) presenting with concomitant vulvar/external genital varicosities. Mean visual analog scale (VAS) and pelvic venous clinical severity score (PVCSS) both decreased during follow-up, technical success was achieved in all patients, and no major procedure-related adverse events were recorded. These findings suggest that an embolization-based strategy may be a practical treatment approach in this specific clinical subgroup.

The main value of the present study is not to challenge the existing larger-scale evidence supporting embolization in PeVD overall, but to provide focused real-world data in a more specific subgroup of women presenting with vulvar/external genital varicosities in addition to pelvic venous symptoms. Recent systematic reviews and larger retrospective studies, including the systematic review by Hanna et al. and the larger cohort reported by Wang et al., have already documented favorable outcomes of embolization-based treatment in broader PeVD or pelvic congestion syndrome populations (4–6). In contrast, fewer reports have specifically described the procedural workflow and short-term outcomes of patients in whom both treatment of pelvic venous reflux and local management of vulvar/external genital varicosities may be clinically relevant (3, 11–13).

Our findings are broadly consistent with previous reports showing symptom improvement after embolization-based treatment for pelvic venous disease (4, 5, 12, 14, 15). Recent reviews have also emphasized that women with vulvar varicosities may represent a clinically relevant subgroup within the spectrum of pelvic venous disorders (1, 12, 16). In addition, prior studies suggest that local sclerotherapy may be useful for symptomatic vulvar varicosities in selected patients (3, 11). In the present series, adjunctive local sclerotherapy was performed during the same interventional session, whereas intraluminal polidocanol foam during ovarian vein embolization was used selectively according to intraprocedural venographic findings, reflecting real-world practice in this subgroup. However, our study was not designed to determine the incremental benefit of adjunctive sclerotherapy beyond ovarian vein embolization alone, and no comparative conclusions regarding superiority can be made.

The observed reductions in VAS and PVCSS indicate clinically relevant symptom improvement after treatment. At the same time, these findings should be interpreted within the scope of the available data. Quality of life was not assessed using a standardized patient-reported outcome instrument, and follow-up imaging was not standardized across patients, both of which remain important considerations in contemporary studies of pelvic venous disorders (1, 12, 13, 16). Accordingly, imaging findings were considered supportive rather than primary outcome measures. With respect to safety, no major procedure-related adverse events, including access-site complications, coil migration, symptomatic thrombosis, allergic reactions, non-target embolization, or unplanned reintervention, were recorded during follow-up (4, 17).

This study has several limitations. First, the sample size was small, and the retrospective single-center design limits generalizability. Second, four screened patients were excluded because of prior intervention or incomplete records, which may have introduced selection bias and limited the generalizability of the findings. Third, treatment was not fully homogeneous because intraluminal polidocanol foam during ovarian vein embolization was not used uniformly in all patients. Fourth, the study lacked a comparison group and therefore could not assess the added value of adjunctive sclerotherapy beyond embolization alone. Fifth, the follow-up duration was relatively short for a chronic and potentially recurrent venous disorder, and longer follow-up is needed to evaluate durability and late recurrence. Finally, anatomical characterization was limited by the retrospective design, because pelvic escape pathways, detailed vulvar reflux mapping, and renal/iliac venous obstruction were not systematically assessed using a dedicated standardized protocol in all patients, and imaging follow-up was not standardized.

Future multicenter prospective studies with larger patient populations, standardized imaging protocols, and longer follow-up are needed to better define patient selection, procedural strategy, and the role of adjunctive local sclerotherapy in this subgroup.

In conclusion, this study describes a single-center retrospective experience of ovarian vein embolization with adjunctive local sclerotherapy in women with PeVD and concomitant vulvar/external genital varicosities. This embolization-based strategy was technically feasible and was associated with short-term symptom improvement in this specific clinical subgroup.

## Data Availability

The datasets generated and/or analyzed during the current study are available from the corresponding author on reasonable request.
